# Mechanical Prediction of Shale Oil Reservoirs Based on Micromechanical Characterization, Machine Learning, and Upscaling Model

**DOI:** 10.3390/nano16191229

**Published:** 2026-09-29

**Authors:** Xiaolong Wan, Jianming Fan, Rui Chang, Jiayi Dai, Ameng Wu, Huiying Tang, Junliang Zhao

**Affiliations:** 1 Research Institute of Exploration & Development, PetroChina Changqing Oilfield Company, Xi’an 710018, China; wanxl_cq@petrochina.com.cn (X.W.); fanjm_cq@petrochina.com.cn (J.F.); chrui_cq@petrochina.com.cn (R.C.); wameng_cq@petrochina.com.cn (A.W.); 2 State Key Laboratory of Oil and Gas Reservoir Geology and Exploitation, Southwest Petroleum University, Chengdu 610500, China; jiayi_dai_swpu@163.com (J.D.); tanghuiying@swpu.edu.cn (H.T.)

**Keywords:** shale oil, nanoindentation, machine learning, Mori–Tanaka model, mechanical upscaling

## Abstract

Shale oil reservoirs exhibit strong heterogeneity and complex rock mechanical properties. Accurate geomechanical modeling is crucial for optimizing horizontal well trajectories and designing fracturing parameters. Traditional mechanical experiments are costly and provide limited data, while empirical formulas suffer from poor regional applicability. Machine learning models designed to predict reservoir mechanical parameters commonly overlook lithofacies-specific variations. To overcome this limitation, this paper presents and adopts a novel workflow that combines micromechanical characterization, machine learning prediction, and mechanical upscaling. Nanoindentation tests are first performed in Well W1 in the Ordos Basin to obtain the mechanical properties of different micro-constituents in shale. Then, a machine learning model is used to establish the relationship between conventional well logs and micro-constituent contents, thereby predicting the distribution of micro-constituents along the entire wellbore. Finally, based on micromechanical derivation, the Mori–Tanaka model is applied for mechanical upscaling to obtain the Young’s modulus. The feasibility of this method is validated by comparing the results with those calculated from conventional well log data. This approach combines the high-precision micromechanical characterization of nanoindentation with the predictive capability of machine learning. It overcomes the limitations of elemental capture spectroscopy logging (namely, its high cost and insufficient organic matter information) and provides a new pathway for establishing one-dimensional geomechanical models.

## 1. Introduction

Shale oil, an unconventional oil and gas resource, plays a significant role in the global energy system. Due to the inherently low porosity and permeability of the reservoir, reservoir enhancement is typically a prerequisite for commercial production [1]. Currently, horizontal drilling and hydraulic fracturing technologies are the predominant methods for shale oil development [2]. However, continental shale oil reservoirs in China are characterized by strong vertical heterogeneity and rapid lithological changes [3], which lead to complex rock mechanical properties in the reservoir and pose challenges for selecting horizontal target zones and predicting hydraulic fracture propagation. Therefore, studying the mechanical properties of shale oil reservoirs is of great significance.

Geomechanics serves as a bridge between geology and engineering, and accurate geomechanical modeling is crucial for selecting wellbore trajectories and determining fracturing parameters [4,5]. Determining the mechanical properties of shale reservoirs through core mechanical experiments is the most direct method [6,7,8]. However, traditional mechanical tests are costly and time-consuming, and limited macroscopic mechanical data have proven insufficient for engineering applications because the micro-constituents of shale vary significantly in the vertical direction [9]. Numerous empirical models have been developed to estimate the relationship between rock mechanics and petrophysical logs [10,11]. The accuracy of these empirical formulas is limited in different regions, which restricts their application. With advancements in computer technology, machine learning (ML) models have increasingly been applied to predict mechanical parameters of whole shale reservoirs [12,13,14]. However, a significant limitation of these models is that they fail to account for differences among lithofacies within shale. Extensive studies have shown that the mechanical properties of shale are influenced by factors such as micro-constituents and microstructure [15]. Different lithofacies within shale exhibit variations in the content of their micro-constituents, resulting in differences in mechanical properties. Furthermore, the alternating distribution of different lithofacies within shale reservoirs leads to even greater complexity in the mechanical properties of these reservoirs.

Zhao et al. [16] and Zhang et al. [17] used elemental capture spectroscopy (ECS) logging information to perform mechanical upscaling at the well scale and validated the feasibility of the upscaling method by comparing it with the dynamic Young’s modulus interpreted from conventional logging. Upscaling models mainly include the Voigt–Reuss–Hill (VRH) method [18], the dilute method [19], and the Mori–Tanaka (MT) model [20]. The VRH model evaluates the elastic modulus of rocks by integrating the elastic behavior of aggregates and single crystals. The dilute method assumes that all mineral constituents are continuous, homogeneous, and linearly elastic, and the effective mechanical properties can be treated as a simple superposition of individual mineral crystal inclusions. Compared with the aforementioned two homogenization approaches, the MT model accounts for inclusion interactions. It independently computes the strain concentration tensor for each phase and then obtains the overall response via volume-fraction-weighted averaging. Moreover, its computational time is substantially lower than that of numerical simulation methods [21], significantly reducing computational cost and making it suitable for multiscale computation and engineering applications.

Within the MT model, the mechanical properties and contents of different micro-constituents are essential. Nanoindentation, modulus mapping, PeakForce quantitative nanomechanical mapping, and contact resonance are currently the most widely used methods for micromechanical characterization [22,23]. Among these methods, nanoindentation has been proven to characterize micromechanical properties more accurately than mechanical mapping methods [24]. For the micro-constituent content of the whole shale reservoir, although ECS logging can provide mineral composition data along the entire wellbore, it lacks information on organic matter content. Meanwhile, the high cost of ECS logging limits its application in most wells.

Predicting micro-constituents based on machine learning models and conventional logging has proven effective [25,26]. Therefore, taking Well W1 in the Ordos Basin as our subject, nanoindentation testing was employed to characterize the mechanical properties of different micro-constituents within shale. A machine learning model was applied to learn the relationship between conventional logging data and composition in the same region, and the content of different micro-constituents was predicted in Well W1. We then performed mechanical upscaling based on the MT model using micromechanical properties obtained from nanoindentation and mineral information derived from machine learning. By comparing the results with the dynamic Young’s modulus obtained from a one-dimensional geomechanical modeling process, we revealed the feasibility of the proposed workflow. This research provides new approaches for establishing one-dimensional geomechanical models.

## 2. Experiment and Methods

### 2.1. Micromechanical Characterization

#### 2.1.1. Shale Sample Preparation

A shale sample used in this study was obtained from the Chang 7 Member of the Yanchang Formation shale oil reservoir in the Ordos Basin, China, at a true depth of 1000 m. The sample was then cut into a 10 × 10 × 5 mm block. The cut sample was polished with sandpaper, and argon ion polishing was used for the nanoindentation experiment.

#### 2.1.2. Micro-Constituent Analysis

Scanning electron microscopy (SEM) and energy-dispersive spectroscopy (EDS) were performed to observe and select the test areas (Figure 1a) and to analyze the elemental distribution (Figure 1b) on the sample surface. Micro-constituents of twenty 20 μm × 20 μm areas were identified and distinguished, facilitating localization of target micro-constituents for the subsequent nanoindentation test.

#### 2.1.3. Nanoindentation Tests

Nanoindentation has been proven to be a powerful technique for characterizing micromechanical properties [27]. A Hysitron Tribo Indenter 900 nanoindentation system was used in this study to characterize the mechanical properties of micro-constituents in shale. A Berkovich indenter made of diamond with a regular three-sided pyramid shape was employed. Before testing, a fused silica sample was used to calibrate indenter parameters. A 5 × 5 indentation grid was performed on its surface under load-controlled mode. During the nanoindentation test, the Optical microscope equipped in the nanoindentation system was used to position the indenter near the selected areas, and scanning probe microscopy (SPM) mapping (Figure 1c) was subsequently performed. Nanoindentation tests were conducted on the main micro-constituents of each region based on the micro-constituents identification results, only after the SPM results matched the corresponding SEM images well. Maximum loads of 3000 μN and 1000 μN were applied to inorganic minerals and organic matter, respectively (Figure 1d). Each indentation consisted of loading (10 s), holding (2 s), and unloading (10 s) stages. The procedures of the nanoindentation test are depicted in Figure 1.

Complete load–displacement curves were recorded from the indenter’s initial contact with the shale sample surface to its withdrawal. According to the Oliver–Pharr methodology [28], the mechanical properties of micro-constituents can be determined as follows:

(1) H=PmaxAc

(2) $$E_{r}=\frac{\sqrt{\pi }}{2\beta \sqrt{A_{c}}}S$$

where H is the hardness; $P_{max}$ is the maximum loading force; $A_{c}$ is the contact area; Er is the reduced modulus; S is the slope of the initial unloading portion of the load–displacement curve; and β is the geometric correction factor. For the Berkovich indenter, β = 1.034.

To obtain the Young’s modulus, the following conversion equation is required:

(3) $$\frac{1}{E_{r}}=\frac{1-\upsilon^{2}}{E}+\frac{1-\upsilon_{i}^{2}}{E_{i}}$$

where υ is the Poisson’s ratio of the micro-constituent; E is the Young’s modulus of the micro-constituent; and $\upsilon_{i}$ and $E_{i}$ represent the Poisson’s ratio and Young’s modulus of the indenter, respectively.

### 2.2. Machine Learning

#### 2.2.1. Data Acquisition and Processing

The data for the machine learning model in this study were collected from the open literature of Lyu et al. [29], including resistivities (RT), natural gamma ray (GR), acoustic (AC), density (DEN), neutron (CNL) logs, and their corresponding micro-constituent content data of Chang 7 Section (118 m thick). The Chang 7 shale consists of quartz, feldspar, chlorite, illite, calcite, dolomite, pyrite, and organic matter. These logging data were resampled with a vertical resolution of 0.125 m, yielding a total of 945 depth datasets. The contents of different micro-constituents at the same depth were normalized according to the following equation:

(4) $$\omega_{in}=\frac{\omega_{i}}{1+\omega_{om}}$$

where $\omega_{in}$ is the normalized weight content of different micro-constituents; $\omega_{i}$ is the weight content of different micro-constituents; and $\omega_{om}$ is the content of organic matter.

Quartz and feldspar were grouped together as QFM, while calcite and dolomite were merged into a carbonate mineral to reduce model prediction complexity. Figure 2 shows the data used for the machine learning model. The responses of different well logging methods to the reservoir are significantly different. Within this 118 m reservoir, the shale exhibits strong heterogeneity, with the QFM content ranging from 8.21% to 80.59%. The variation ranges of carbonate, clay, and pyrite are 0.08–55.59%, 0.85–13.90%, and 0–34.45%, respectively. The mean content of organic matter is 14.69%. The conventional well logging shows significant fluctuations below 2050 m.

The StandardScaler method was employed in this study to transform five conventional logging parameters, which have different units, into a distribution with a mean of 0 and a variance of 1. This allows the machine learning model to learn the importance of each feature impartially, thereby enhancing the model’s robustness. The formula is as follows:

(5) $$X_{scaled}=\frac{X-\mu }{\sigma }$$

where $X_{scaled}$ is the conventional logging data after StandardScaler; X is the original conventional logging data; μ is the mean of the original conventional logging data; and σ is the variance of the original conventional logging data.

#### 2.2.2. Micro-Constituent Prediction Model

Hu et al. [25] developed a ConvXGB model to predict inorganic mineral contents, showing high accuracy compared with the geochemical logging data and X-ray Diffraction (XRD) experimental results. Given that XGB is a tree-based model with numerous parameters, and that the process of optimizing parameters is complex and time-consuming, only the Conv architecture for model training and prediction is retained in this work. Other studies have demonstrated the efficiency of Conv models [30,31]. The Conv model is a Convolutional Neural Network (CNN) with an encoder–decoder architecture (Figure 3). The downsampling path on the left side is an encoder, consisting of a convolutional layer with a 3 × 3 kernel, max-pooling with a 2 × 2 stride, and a sigmoid activation function, resulting in a reduction in feature map size and an increase in the number of feature channels. This allows the model to capture high-level information. The upsampling path on the right side is the decoder, including up-convolution, skip concatenation with the corresponding features from the downsampling path, and transposed convolution with the same kernel and stride. The spatial resolution is gradually recovered through these operations. The dropout rate along the whole path is set to 0.5. The resulting features are then passed through a single-channel convolutional layer and a flattening layer, and fed into two fully connected layers. It is worth noting that before the conventional logging data were input into the model, data augmentation was performed by simply replicating the data to improve model performance.

#### 2.2.3. Model Establishment

A total of 945 data sets were shuffled and subdivided into a training set (800) and a test set (145). The training set was further divided into training and validation subsets at a ratio of 4:1. The conventional logging data were used as model input, with micro-constituent contents as labels for model training and prediction. The root mean square error (RMSE) was employed as the loss function to quantify the discrepancy between model predictions and actual values:

(6) $$RMSE=\sqrt{\frac{\sum_{i=1}^{n}{\left(y_{act}-y_{pre}\right)}^{2}}{n}}$$

where $y_{act}$ is the actual value, and $y_{pre}$ is the prediction value of the model.

Meanwhile, the Adam optimizer and an early-stopping strategy were employed, thereby improving both training speed and stability of the model.

### 2.3. Lithological Classification Upscaling Model

#### 2.3.1. Mori–Tanaka Upscaling Method

Within the framework of composite micromechanics and on the basis of the equivalent homogenization concept [32], the MT model is employed to predict an equivalent homogeneous medium with macroscopic mechanical behavior consistent with that of multiphase mineral aggregate units [20] (Figure 4).

Based on the linear elastic constitutive relation, for a composite material containing M phases, let the volume fraction of an arbitrary *r*-th phase be $V_{r}$. The macroscopic elastic modulus of the equivalent medium can then be expressed as

(7) $$C_{hom}=\sum_{r}V_{r}C_{r}:A_{r}$$

where $A_{r}$ is the strain concentration tensor of the *r*-th phase.

Therefore, estimating the macroscopic stiffness tensor of multiphase materials hinges on calculating the strain concentration tensor. Consider the *r*-th phase placed in a medium of stiffness $C^{0}$ and subjected to a uniform strain $E^{0}$ at infinity; the strain generated in this phase is

(8) $$\varepsilon_{r}={\left[I+P:(C_{r}-C^{0}\right)}^{-1}:E^{0}]$$

where I is the fourth-order identity tensor, and P is the Hill tensor, which depends on the shape of the inclusion phase and the stiffness of the aforementioned medium. Laws [33] proposed expressions for the non-zero components when spheroidal inclusions are embedded in a transversely isotropic medium. In the present study, inclusions are all considered spherical, and thus the aspect ratio is taken as 1.

By incorporating the average strain of the multiphase mineral aggregate, the expression for the strain concentration tensor is obtained as

(9) $$A_{r}={\left[I+P_{r}^{0}:(C_{r}-C^{0})\right]}^{-1}:{\left\{\sum_{r}V_{r}{\left[I+P_{r}^{0}:(C_{r}-C^{0})\right]}^{-1}\right\}}^{-1}$$

where, according to the MT model concept, $C^{0}$ is denoted as $C_{0}$, which represents the stiffness tensor of the matrix phase.

#### 2.3.2. Lithofacies-Based Mechanical Property Prediction Scheme

In the derivation of the aforementioned upscaling method, it is evident that predicting the macroscopic mechanical properties of multiphase mineral aggregates requires the initial determination of the stiffness of the matrix phase, $C_{0}$. Ogierman [34] pointed out that when the MT model is applied to systems with a high volume fraction of inclusion phases, close-range interactions among inclusions are significantly enhanced. However, the mean-field approximation inherent to the MT method cannot fully capture such effects. Therefore, the volume fraction of each mineral phase within the aggregate is a critical controlling factor. Within the depth interval under investigation, two main lithofacies (sandstone and mudstone) can be broadly distinguished based on geophysical well log interpretations. Thomsen [35] noted that mineral preferred orientation is a significant reference value. Considering these factors, we account for the supporting role of quartz minerals in the sandstone framework and their influence on failure behavior [36,37], as well as the dominant role of clay minerals in the macroscopic mechanical properties of shale [38], when predicting mineral composition. Consequently, a targeted approach is proposed, wherein the initial matrix phase for the upscaling model is selected according to lithofacies classification.

Assuming quartz to be isotropic, the stiffness matrix consists of only two independent constants, and the calculation formula is as follows:

(10) $$C_{quartz}=\left[\begin{array}{cccccc} \eta +2\chi & \eta & \eta & & & \\ \eta & \eta +2\chi & \eta & & & \\ \eta & \eta & \eta +2\chi & & & \\ & & & \chi & & \\ & & & & \chi & \\ & & & & & \chi \end{array}\right]$$

(11) $$\chi =\frac{E_{q}}{2(1+\upsilon_{q})}$$

(12) $$\eta =\frac{E_{q}\upsilon_{q}}{\left(1+\upsilon_{q})(1-2\upsilon_{q}\right)}$$

where $E_{q}$ is the Young’s modulus of quartz, and $\upsilon_{q}$ is the Poisson’s ratio of quartz.

The stiffness matrix of clay is adopted from the research of Zhao et al. [16], and the elastic modulus of quartz is obtained from field experiments. The mineral stiffness matrix parameters utilized in this study are listed below in Table 1.

In the model presented in this paper, non-clay/quartz minerals, organic matter, and pores are treated as isotropic spherical inclusions, which are assumed to be relatively uniformly distributed within the matrix phase. Meanwhile, the effects of microcracks and fluids on the macroscopic prediction results are not considered. To simplify the model prediction workflow, carbonate minerals are grouped together, while stiff minerals (e.g., feldspar, pyrite) are considered as a separate class. The equivalent elastic moduli of the two groups are then obtained using volume-weighted averaging (for carbonates) and the Reuss lower bound (for hard minerals) [39], respectively, before being incorporated into the calculation.

During upscaling, a lithofacies-based two-phase inclusion approach is adopted in this paper (Figure 5). Mineral phases are introduced in order of increasing mechanical strength. First, pores are incorporated into the initial matrix phase according to their volume fraction, and the mechanical properties of the equivalent homogeneous medium formed by the two constituents are calculated using an upscaling model. This equivalent homogeneous medium is then taken as the new matrix phase, and the next inclusion phase is incorporated according to its volume fraction. This process is repeated until all inclusion phases have been successively incorporated.

Using this procedure, the stiffness of the equivalent homogeneous medium can be expressed as

(13) $$C_{hom}=V_{0}C_{0}:A_{0}+V_{1}C_{1}:A_{1}$$

where $V_{0}$ and $V_{1}$ are the volume fractions of the matrix phase and the inclusion phase, respectively; $A_{0}$ and $A_{1}$ are the strain concentration tensor of the matrix phase and the inclusion phase, respectively.

## 3. Results

### 3.1. Mechanical Properties of Micro-Constitutes

A total of 157 indentations were performed on the surfaces of pyrite, quartz, feldspar, dolomite, clay, calcite, and organic matter across 20 different areas, with 18, 23, 29, 8, 50, 10, and 19 indentations, respectively. Figure 6a shows the load–displacement curves of these indentations. When the same maximum load was applied to different inorganic minerals, their maximum displacements exhibited various responses, with pyrite showing the lowest maximum displacement and clay displaying the widest variation in maximum displacement. Although the maximum load for organic matter was set to only 1000 μN, its maximum depth falls between those of calcite and clay. The statistical analysis results of Young’s modulus and hardness derived from these load–displacement curves using Equations (1)–(3) are presented in Figure 6b. The Poisson’s ratios used in this work for different micro-constituents are shown in Table 2. The micromechanical properties of the seven micro-constituents exhibit significant discrepancies, demonstrating the strong micromechanical heterogeneity of shale. Among these micro-constituents, pyrite exhibits the highest Young’s modulus, approximately 135.48 GPa with a standard deviation of 47.81, followed by dolomite and quartz, with Young’s modulus values of 107.75 GPa (±14.49) and 71.62 GPa (±12.13), respectively. The average Young’s modulus of feldspar is comparable to that of calcite, with values of 60.95 GPa (±9.01) and 60.45 GPa (±12.40), respectively. The Young’s modulus values of clay and organic matter are 21.36 GPa (±6.87) and 9.13 GPa (±2.03), respectively. Table 3 compares the micromechanical characterization results obtained in this study with those reported in previous studies [40,41]. The results are generally consistent, and the microscopic composition characterized in this study is comprehensive.

### 3.2. Performance of Learning Machine Model

After 100 epochs of training, the performance of the Conv model on the test set is shown in Figure 7. The model demonstrates good predictive ability, with R^2^ exceeding 0.65 for all five categories of micro-constituents defined in this study. QFM and pyrite have the highest R^2^ values, both at 0.86. The R^2^ of clay is 0.71, while those for carbonate minerals and organic matter are 0.68 and 0.66, respectively. The relatively lower prediction performance for these micro-constituents may be attributed to the limited training data.

The micro-constituent contents of the reservoir formation in Well W1 from 1897.8 m to 2179.7 m were predicted by applying the trained Conv model to the conventional logging data, as shown in Figure 8. According to the prediction, there are significant variations in micro-constituent contents at certain depths. QFM and clay are the main minerals in Well W1, and the average content of carbonate minerals is around 18%. Pyrite content is relatively low in the shale reservoir of Well W1, with a maximum of 11%. The organic matter content ranges from 0.2% to 14.3% (Figure 8).

### 3.3. Macroscopic Mechanical Properties

Since the detection scale of nanoindentation is much smaller than the characteristic size of pores and fractures, the measurement process can effectively avoid defect interference, and the obtained modulus essentially reflects the intrinsic elastic response of the mineral matrix. Similarly, the dynamic Young’s modulus is based on high-frequency vibration measurements, in which elastic waves tend to bypass defects during propagation, and thus also reflects the near-matrix stiffness. Nanoindentation and the dynamic Young’s modulus are therefore comparable in terms of their insensitivity to defect-induced weakening, which explains why the rock Young’s modulus derived from nanoindentation converges toward the dynamic value. In contrast, the static modulus fully reflects the softening effects of pores and fractures under slow loading, and thus exhibits a systematic deviation from the nanoindentation results. Kumar et al. fitted the nanoindentation modulus and dynamic modulus of 61 shale samples and found good agreement between them, which further demonstrates that the modulus determined by nanoindentation tends toward the dynamic result [43]. The mineral modulus input data adopted in the upscaling model in this paper are nanoindentation test results, which makes the predicted values tend toward the dynamic modulus. Therefore, in the subsequent validation presented in this paper, the dynamic modulus is used for comparative analysis.

Using geophysical well logging data, the dynamic formation modulus $E_{dyn}$ can be obtained from compressional and shear slowness (AC/DTS) and DEN (Figure 9).

(14) $$V\left(m/s\right)=\frac{1}{\Delta t\left(\mu s/m\right)}10^{6}(\mu s/s)$$

(15) VP(0°)=106ACVs(0°)=106DTS

(16) G=ρVs2109

(17) $$K=\rho (V_{P}^{2}-\frac{4}{3}V_{s}^{2})/10^{9}$$

(18) $$E_{dyn}=\frac{9KG}{\left(3K+G\right)}$$

where AC and DTS are the compressional and shear slowness logs, respectively; $V_{P}$ and $V_{S}$ are the compressional and shear wave velocities perpendicular to the bedding plane, respectively; G is the shear modulus; K is the bulk modulus; and $E_{dyn}$ is the dynamic formation modulus.

The dynamic modulus $E_{dyn}$ calculated from conventional well logging data, the prediction $E_{pre}$ from the lithofacies-based upscaling model proposed in this paper, and the prediction $E_{pre\_Clay}$ from the model considering only clay as the initial matrix phase are compared, as shown in Figure 10.

## 4. Discussion

The nanoindentation test results indicate that the Young’s modulus of different micro-constituents can vary by more than an order of magnitude (pyrite ≈ 135 GPa, organic matter ≈ 9 GPa), which is primarily attributed to inherent differences in mineral crystal structure, chemical bond types, and pore development. The high modulus of pyrite is related to its dense isometric crystal system and strong covalent bonding; quartz and other framework silicates also exhibit high stiffness. In contrast, clay minerals show significantly lower modulus due to their layered structure, while organic matter displays pronounced compliance owing to its amorphous nature and high porosity. It is noteworthy that clay exhibits the widest range of maximum displacement variation, which may reflect the heterogeneous orientation of its internal plate-like particles or the non-uniform distribution of micropores.

In terms of machine learning prediction, although the model achieves R^2^ values exceeding 0.65 for all five constituent categories, prediction accuracy for quartz and pyrite (R^2^ = 0.86) is markedly higher than that for carbonate minerals and organic matter (R^2^ ≈ 0.67). This discrepancy is not attributable to insufficient model generalization but rather to issues related to the distribution of training samples and the discriminability of well-log responses. Conventional log responses (e.g., GR, DEN, AC) for clay and organic matter often overlap, and these constituents commonly occur as mixed phases in actual cores, limiting the representativeness of labeled samples. By contrast, pyrite and quartz, being hard minerals, usually dominate in sandstone intervals and exhibit stable log responses, making them more readily captured by the model. Moreover, organic matter shows the lowest prediction accuracy (R^2^ = 0.66); besides the limited sample size, this may also result from its frequent nano-scale association with clay, its small volume fraction, and its weak contribution to macroscopic logging signals, which hinder the model from isolating its independent signature.

These prediction results have significant practical implications for reservoir evaluation. In Well W1, quartz and clay are the dominant minerals, with carbonate content averaging approximately 18% and organic matter content varying considerably (0.2–14.3%), indicating strong vertical heterogeneity in brittleness index and organic matter abundance within this shale interval. Intervals with high content of rigid minerals (dominated by quartz) are likely to exhibit better fracability, whereas those enriched in clay and organic matter tend to show enhanced plasticity. These findings provide a micro-mechanical basis for subsequent staged fracturing design in horizontal wells. Furthermore, the model enables the acquisition of continuous, high-resolution mineral composition profiles using only conventional well logs in the absence of core XRD data, offering an efficient technical approach for well-to-well correlation and reservoir modeling.

In mechanical upscaling modeling, the mechanical treatment of mineral inclusion phases directly affects the validity of equivalent medium predictions. The present model simplifies the mineral inclusion phases by treating them as isotropic. Observations from image analysis indicate that individual minerals differ significantly in morphology and arrangement. For crystalline minerals, grain orientations are mostly irregular. Although an individual grain may be anisotropic, spatial averaging over a sufficiently large statistical volume cancels out the anisotropic effects; therefore, the grains can be equivalently represented as isotropic spherical inclusions. Clay minerals, however, mostly exhibit platy or layered structures and tend to become preferentially oriented due to processes such as sedimentary compaction. This results in markedly different mechanical properties parallel and perpendicular to the bedding direction, indicating pronounced anisotropy; thus, a transversely isotropic model must be used to describe them (Figure 11). On this basis, for lithofacies-specific prediction, the sandstone facies dominated by crystalline minerals can be modeled as isotropic, whereas the mudstone facies rich in preferentially oriented clay minerals should be modeled as transversely isotropic. This approach not only respects the microfabric characteristics revealed by image analysis but also balances representation accuracy and model efficiency in upscaling calculations.

In actual shale formations, organic matter often occurs as lenticular bodies or bands. For a fixed mineral composition ratio, the influence of the shape factor (aspect ratio) on the predicted values was examined. The results indicate that the morphology of organic matter has only a limited effect on the predictions (Figure 12); therefore, the organic matter phase can be approximately represented as spherical inclusions in the upscaling model.

Using the calculation results derived from conventional well-logging data as a benchmark, the predicted values obtained from the proposed lithofacies-based upscaling model achieve good agreement, with an average relative error of only 6.35% across the entire depth interval. In comparison, the model that solely uses clay as the initial matrix phase yields significant deviations (as exemplified by the area within the green dashed box in Figure 10), which are particularly pronounced in the sandstone lithofacies, leading to an average relative error of 16.65% throughout the studied section. Previous studies have mainly focused on the influence of anisotropy on the mechanical characteristics of shale reservoirs. Nevertheless, the model results reveal that prediction accuracy for the vertical dynamic formation modulus is positively correlated with the dominant mineral matrix properties of each lithofacies. In actual field development, sandstone and mudstone formations exhibit markedly different mechanical behavior. Accordingly, it is essential to incorporate macroscopic mechanical behavior controlled by dominant minerals within corresponding lithofacies during parameter prediction, even though this approach reduces the role of formation anisotropy in mechanical heterogeneity. Meanwhile, the proposed model enables continuous prediction of mechanical parameters along the formation vertical direction (i.e., the well-axis direction of vertical wells), realizing effective prediction at the well scale.

From an engineering perspective, the lithofacies-aware modulus profile provides a continuous description of stiffness variations along the wellbore. For horizontal well placement, it can be used to screen mechanically more uniform landing intervals and identify abrupt modulus contrasts that should be considered during trajectory and completion planning. For hydraulic fracturing design, the modulus profile, when interpreted together with mineral composition, can support stage zoning, cluster placement, and differentiated treatment design. Intervals dominated by rigid minerals such as quartz generally have greater fracability potential, whereas intervals enriched in clay and organic matter tend to exhibit more compliant or plastic behavior. Compared with conventional empirical log-based models, which estimate mechanical parameters without explicitly representing the controlling mineral phases, the proposed workflow links modulus variations to machine-learning-predicted mineral composition, nanoindentation-derived constituent properties, and lithofacies-specific mechanical upscaling. It therefore provides a more physically interpretable representation of mechanical heterogeneity and avoids applying a uniform mechanical model to sandstone and mudstone intervals.

## 5. Conclusions

This study proposes a novel workflow integrating micromechanical characterization, machine learning, and the Mori–Tanaka homogenization scheme to predict the macroscopic Young’s modulus of shale oil reservoirs along the wellbore, using conventional well logs as input. The main conclusions are as follows:

Nanoindentation tests on the Chang 7 shale reveal significant mechanical heterogeneity among seven micro-constituents. The measured Young’s moduli range from ~9 GPa for organic matter to ~135 GPa for pyrite, providing a reliable database for upscaling.

A convolutional neural network (Conv) model trained on conventional logs (GR, RT, AC, DEN, CNL) successfully predicts the vertical distribution of five mineral groups and organic matter. The model achieves R^2^ values above 0.65 for all categories and reaches 0.86 for QFM and pyrite, demonstrating its capability to replace costly ECS logging while also capturing organic matter content.

The MT model, with the initial matrix phase selected according to lithofacies (quartz for sandstone, clay for mudstone), yields predicted Young’s moduli that match the dynamic modulus derived from conventional sonic logs with an average relative error of only 6.35% over the entire depth interval. In contrast, a single-matrix (clay-only) model gives an average error of 16.65%, highlighting the critical role of lithofacies-controlled dominant minerals in upscaling accuracy.

The proposed workflow overcomes the limitations of traditional empirical formulas and expensive specialized logging. It enables cost-effective, data-driven construction of 1D geomechanical models that can guide horizontal well trajectory optimization and hydraulic fracturing design in heterogeneous continental shale reservoirs.

## Figures and Tables

**Figure 1:**
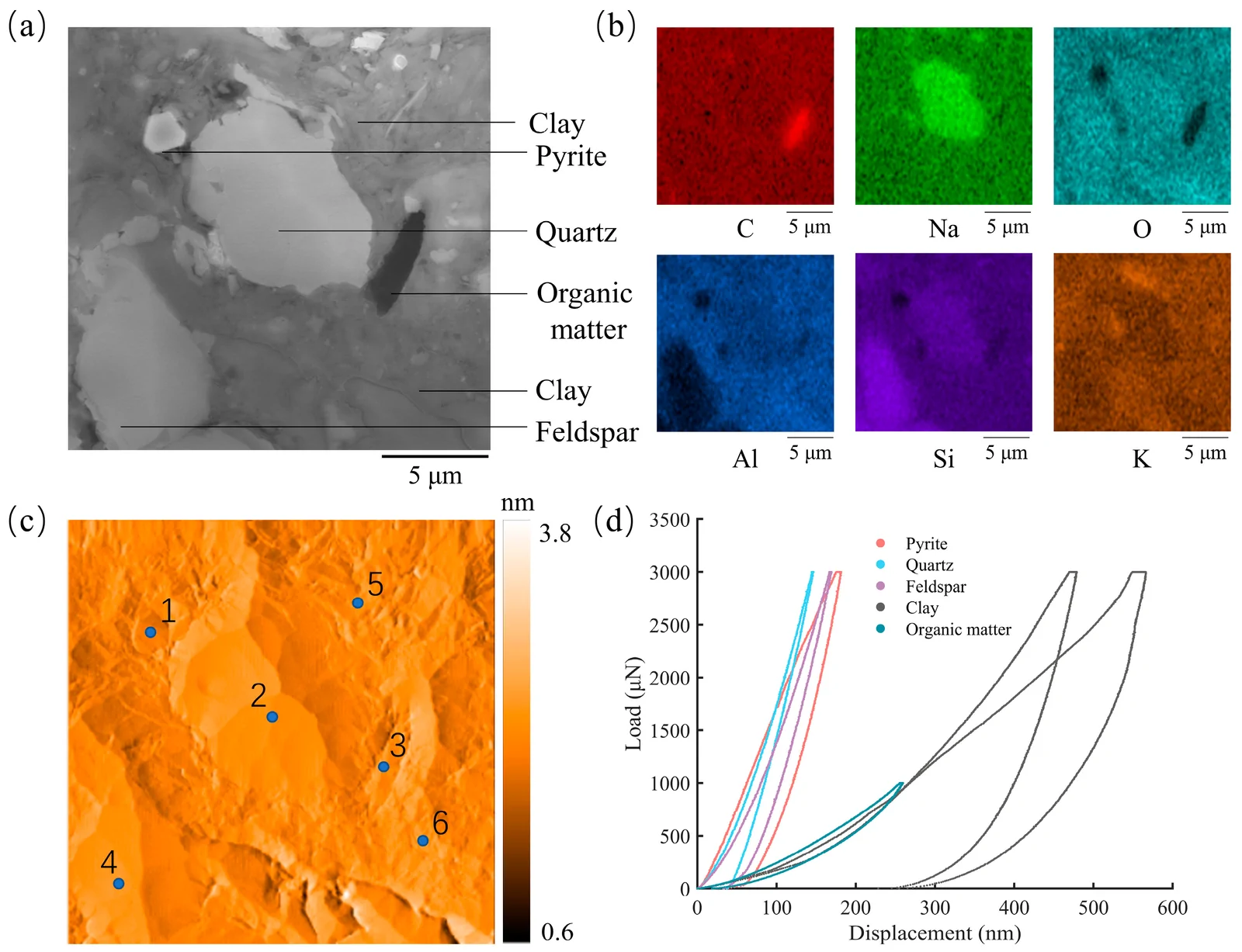
Schematic diagram of the nanoindentation test. (**a**) SEM; (**b**) EDS; (**c**) SPM; (**d**) nanoindentation.

**Figure 2:**
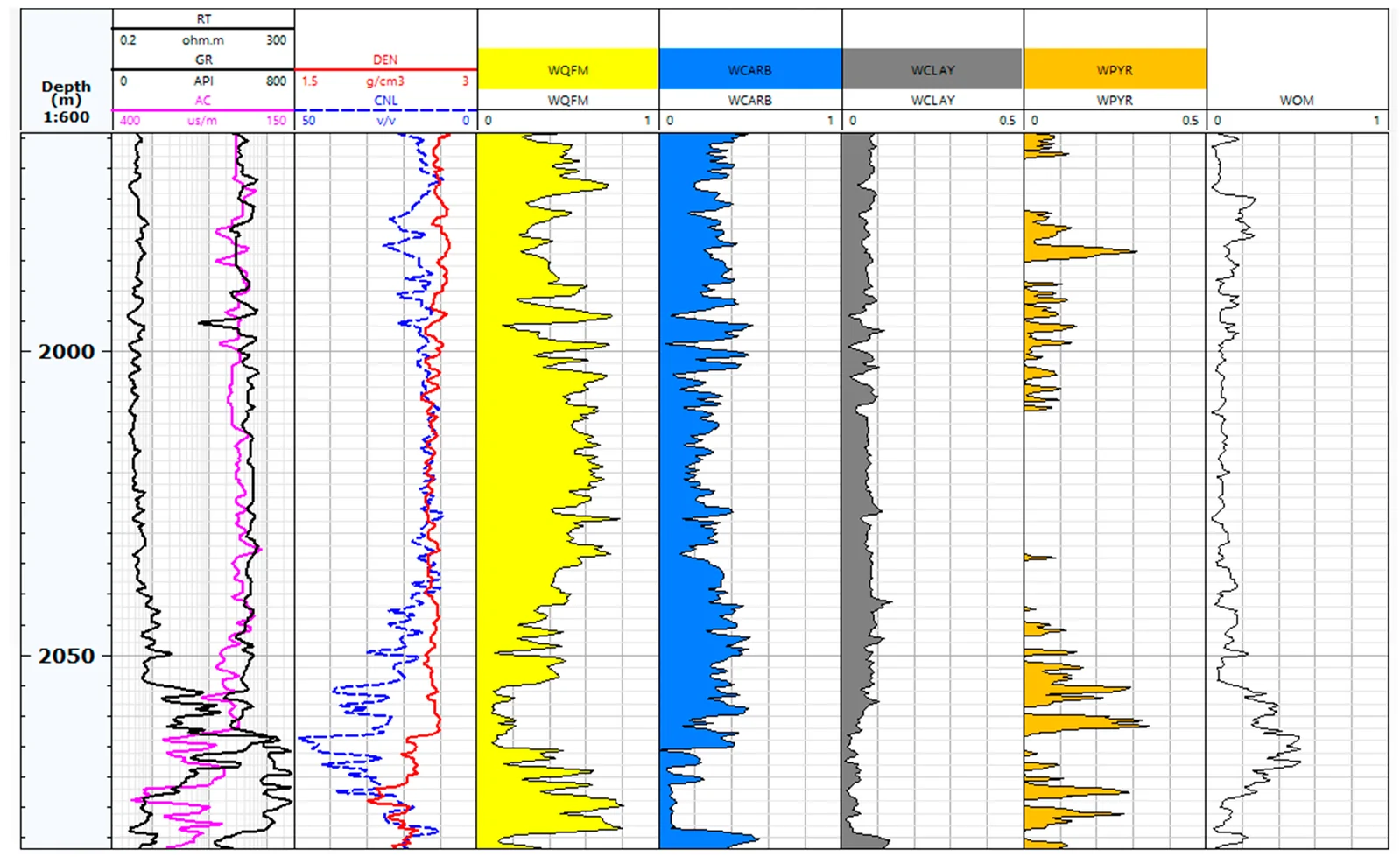
Data used for the machine learning model.

**Figure 3:**
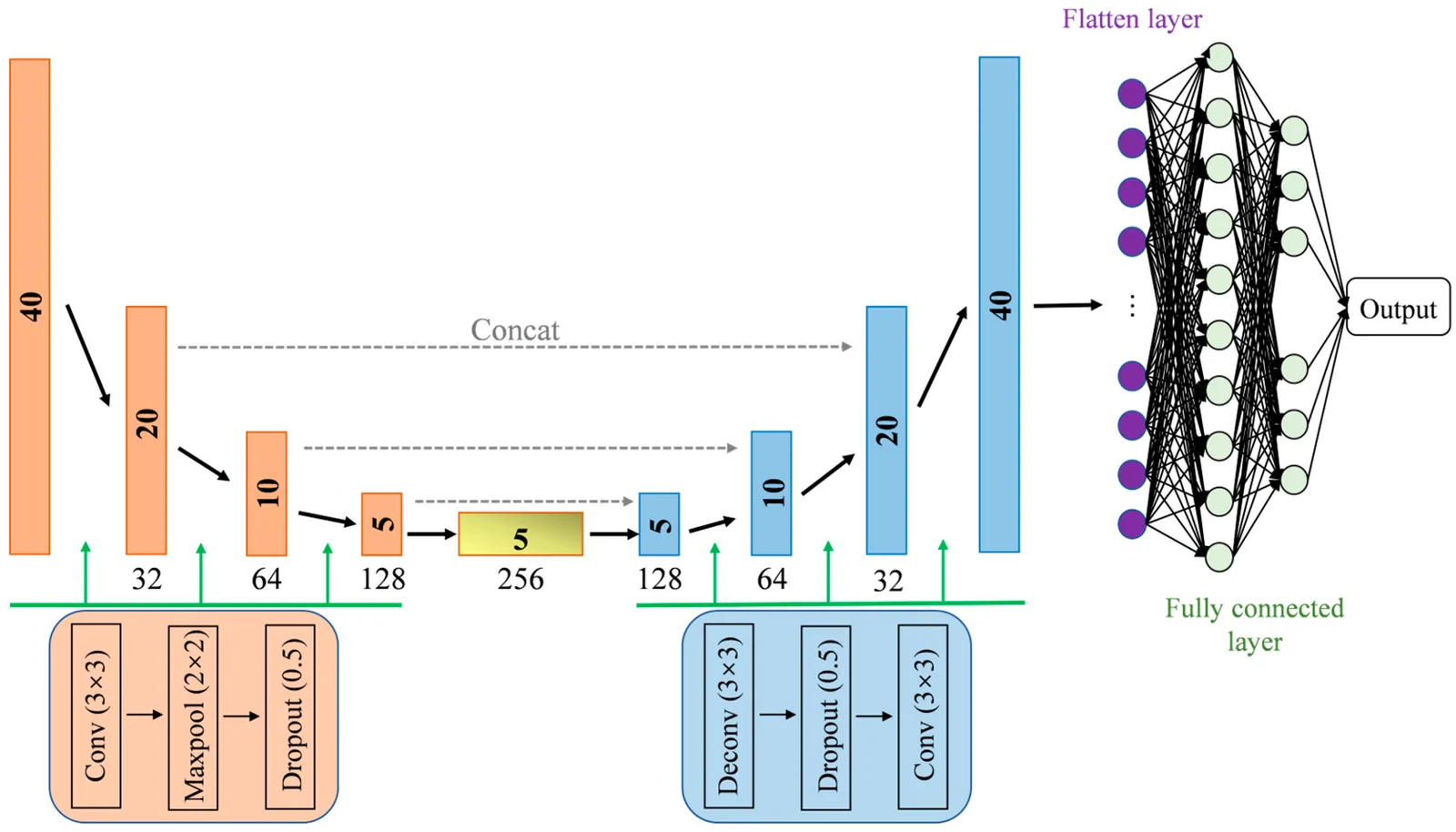
Architecture of the Conv model.

**Figure 4:**
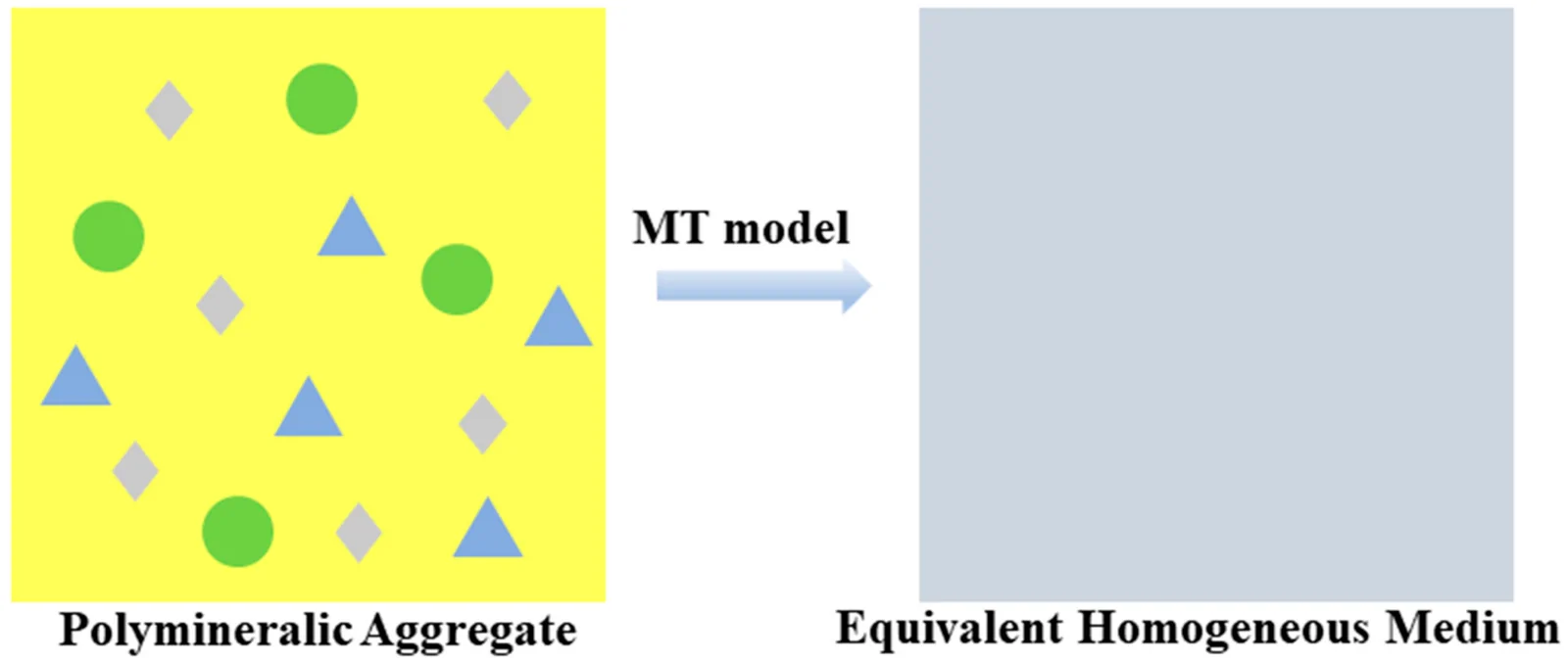
Conceptual diagram of the upscaling mode.

**Figure 5:**
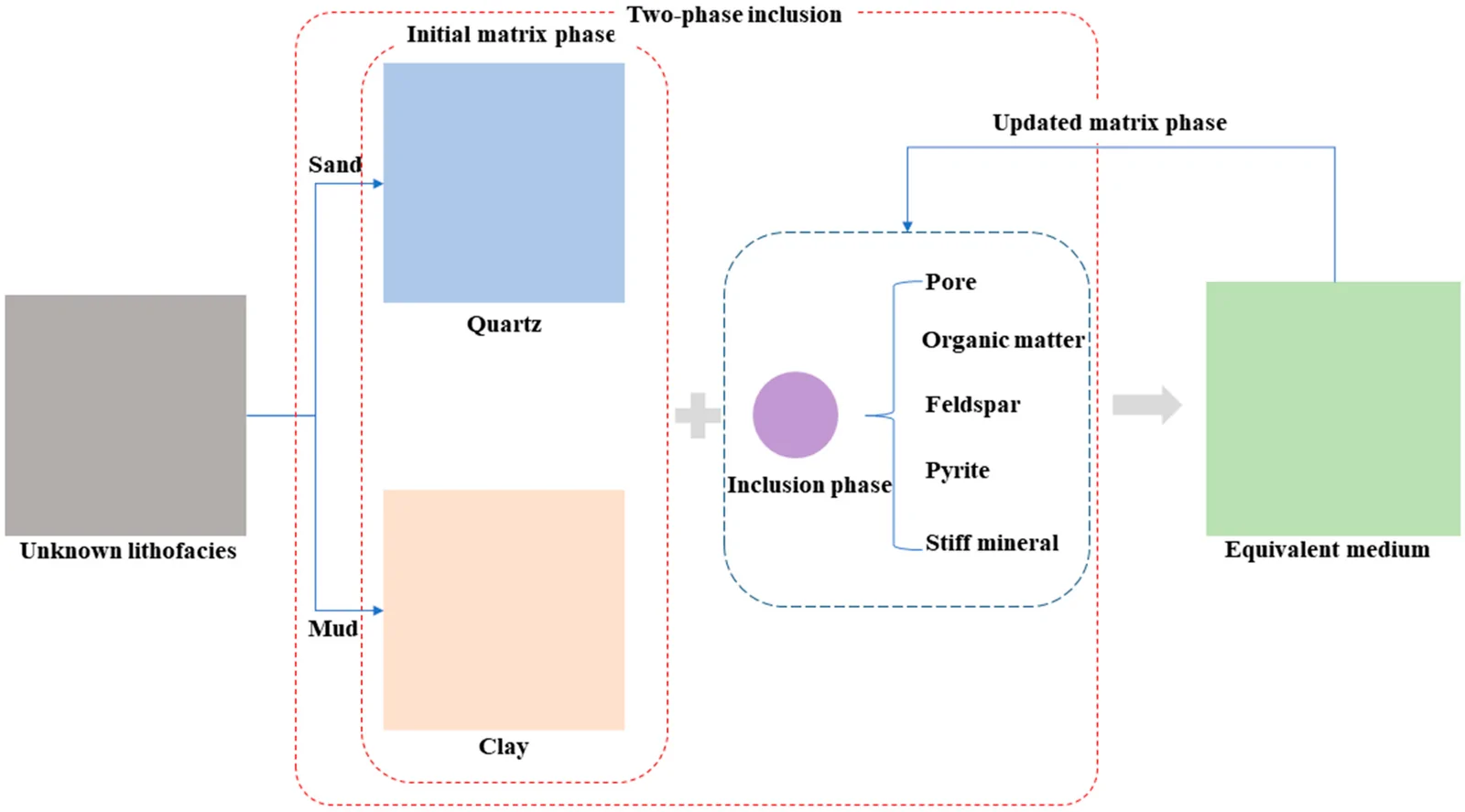
Flowchart of the lithofacies-determined two-phase inclusion scheme.

**Figure 6:**
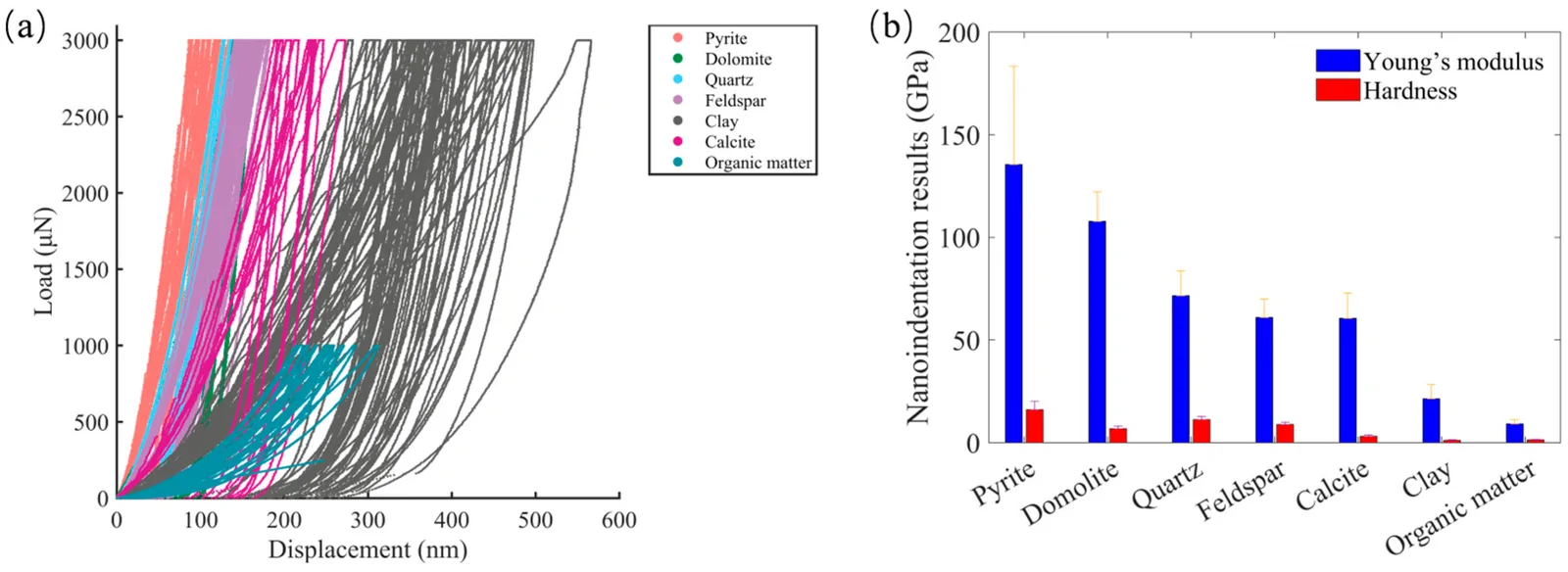
Nanoindentation results. (**a**) Load–displacement curves and (**b**) micromechanical properties.

**Figure 7:**
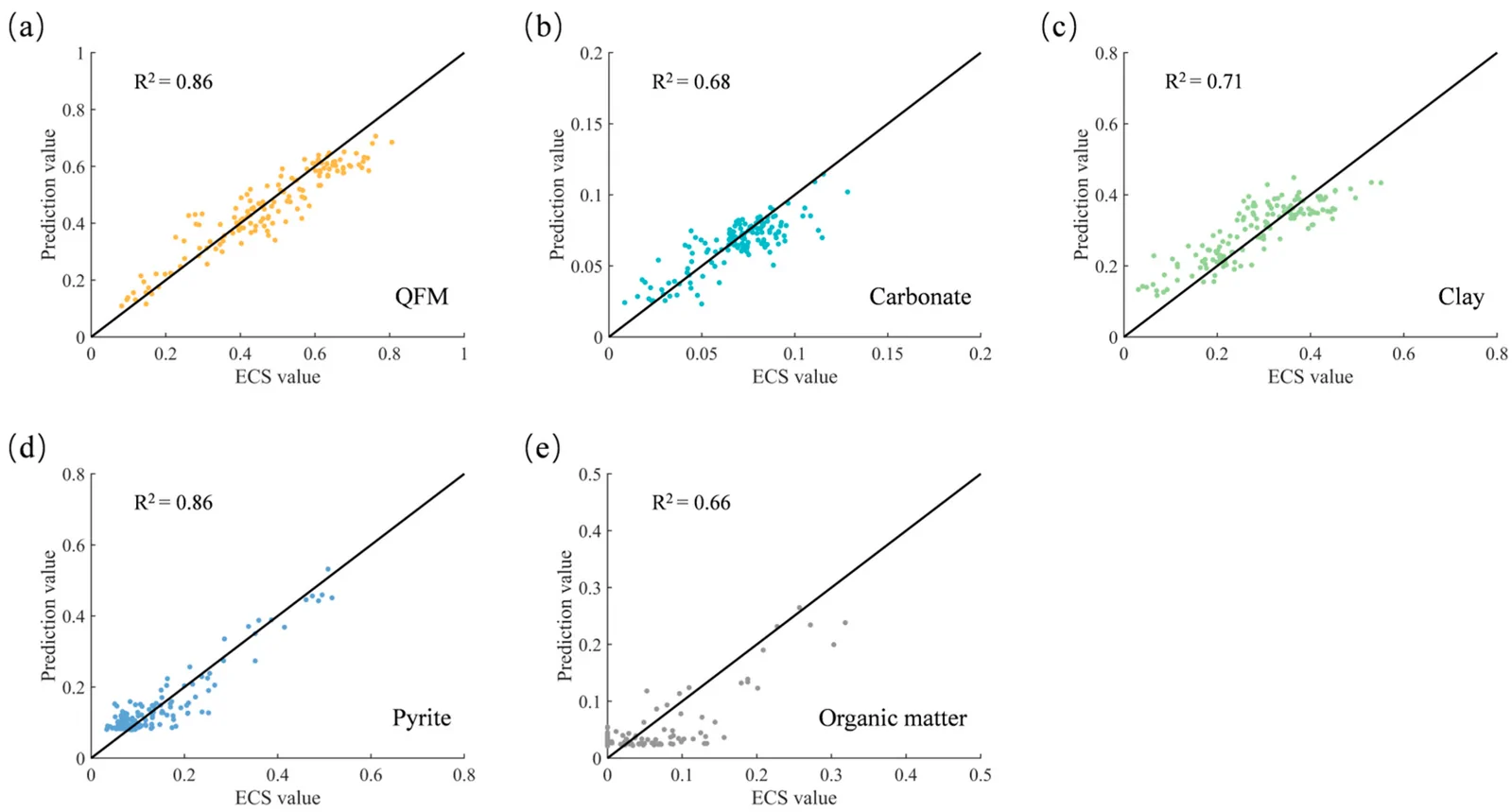
Model performance on the test set: (**a**) QFM; (**b**) Carbonate; (**c**) Clay; (**d**) Pyrite; (**e**) Organic matter.

**Figure 8:**
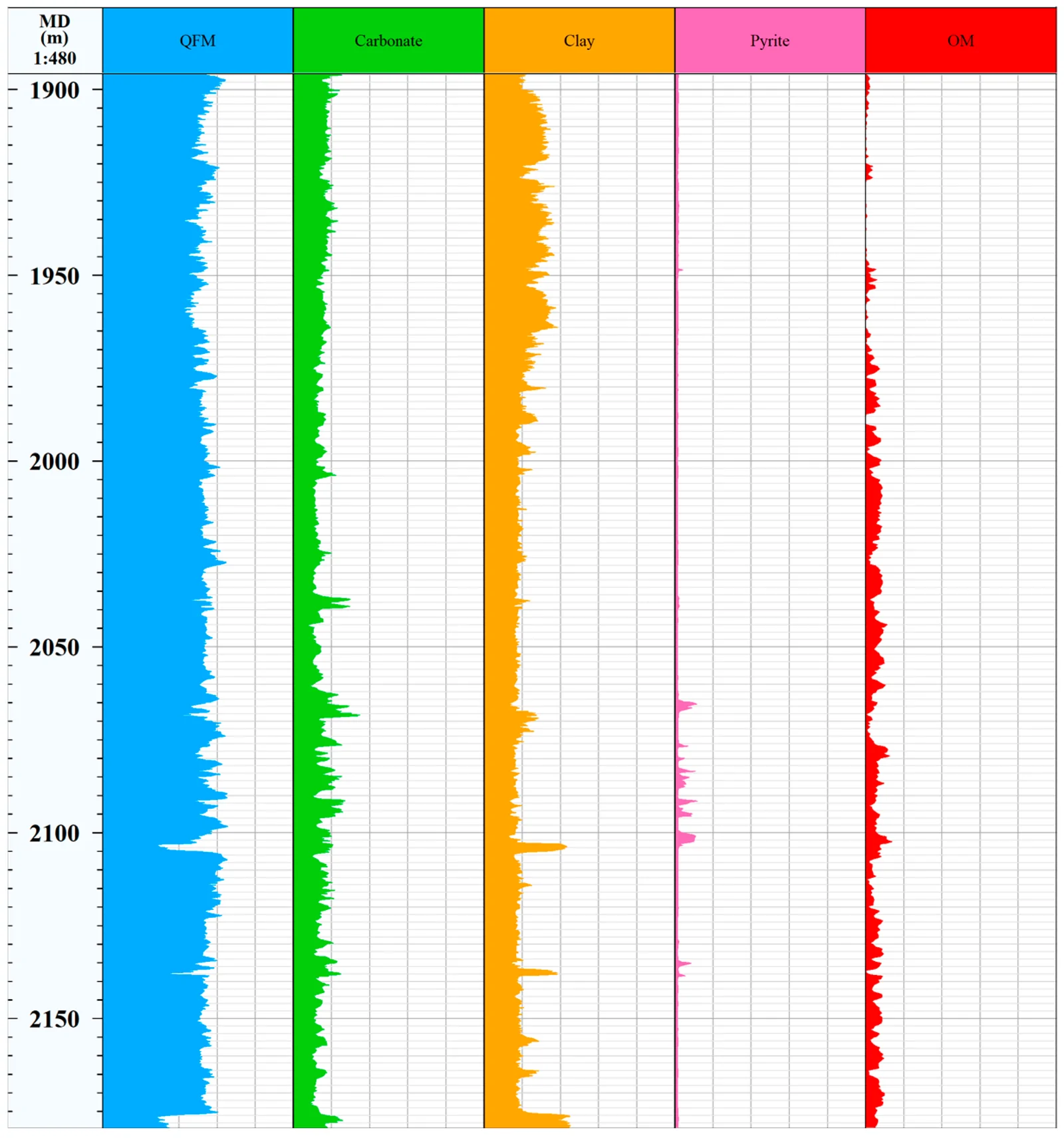
Model prediction for Well W1.

**Figure 9:**
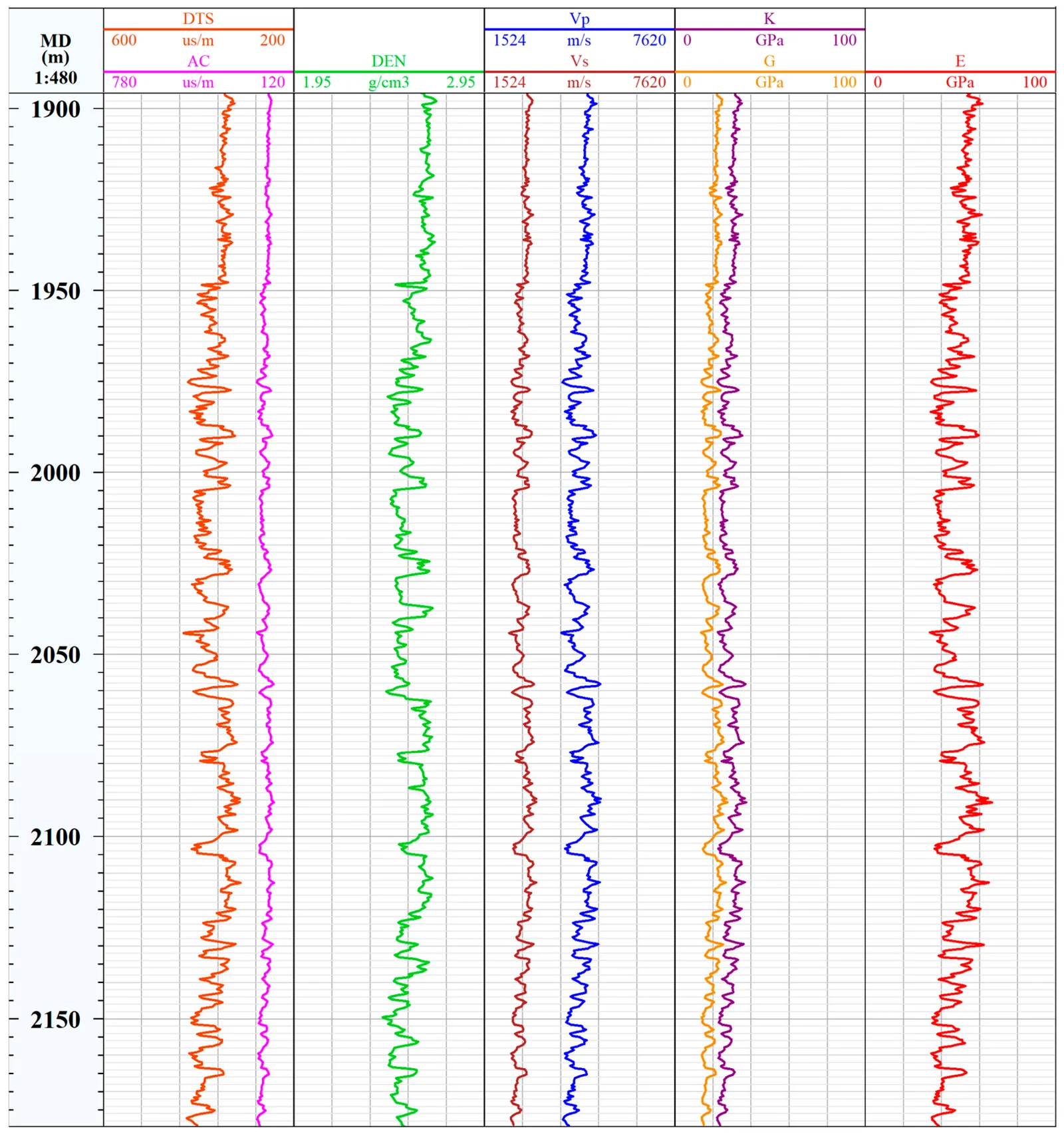
Conventional well logging data and calculated modulus results.

**Figure 10:**
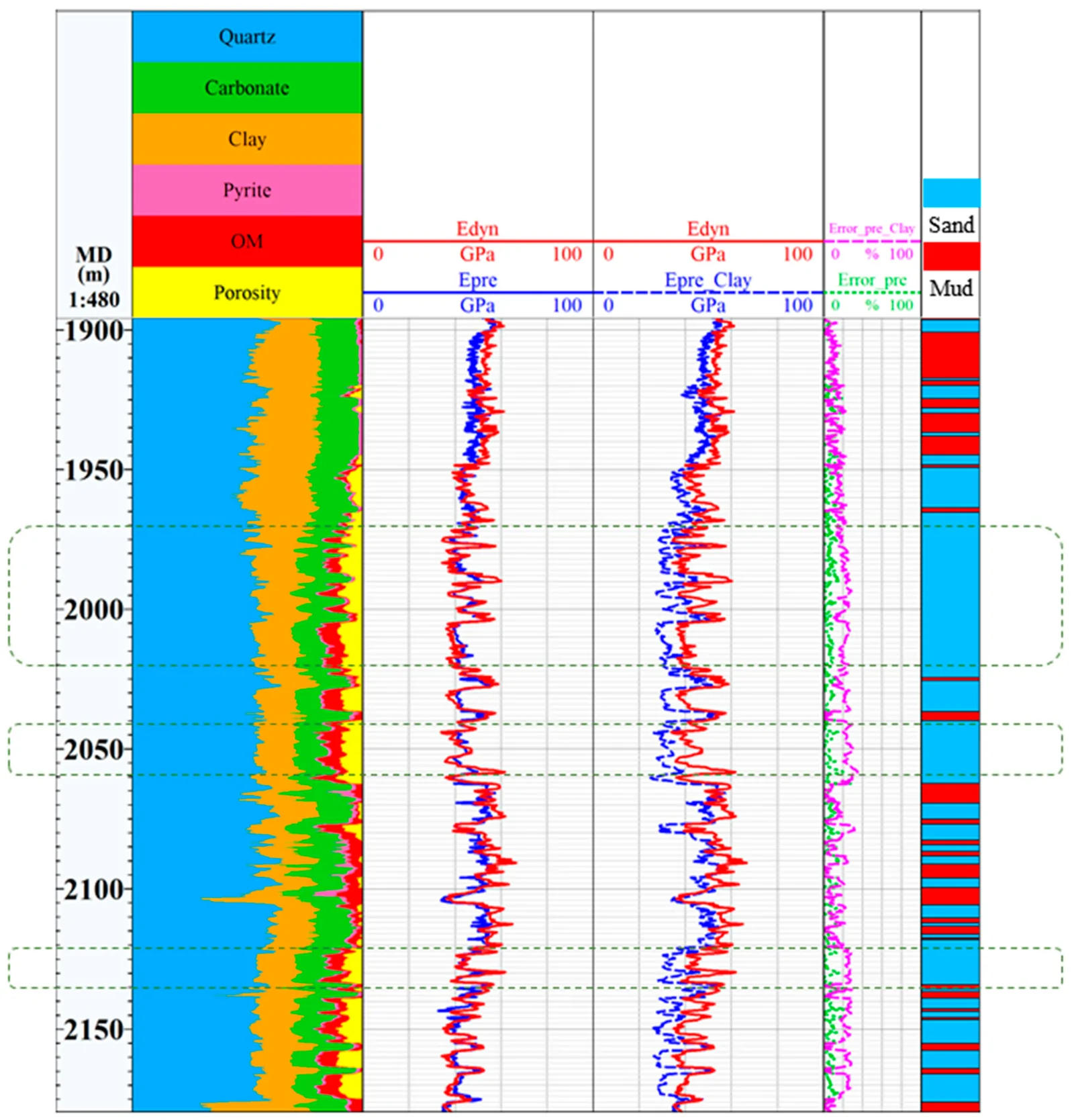
Comparison of prediction results.

**Figure 11:**
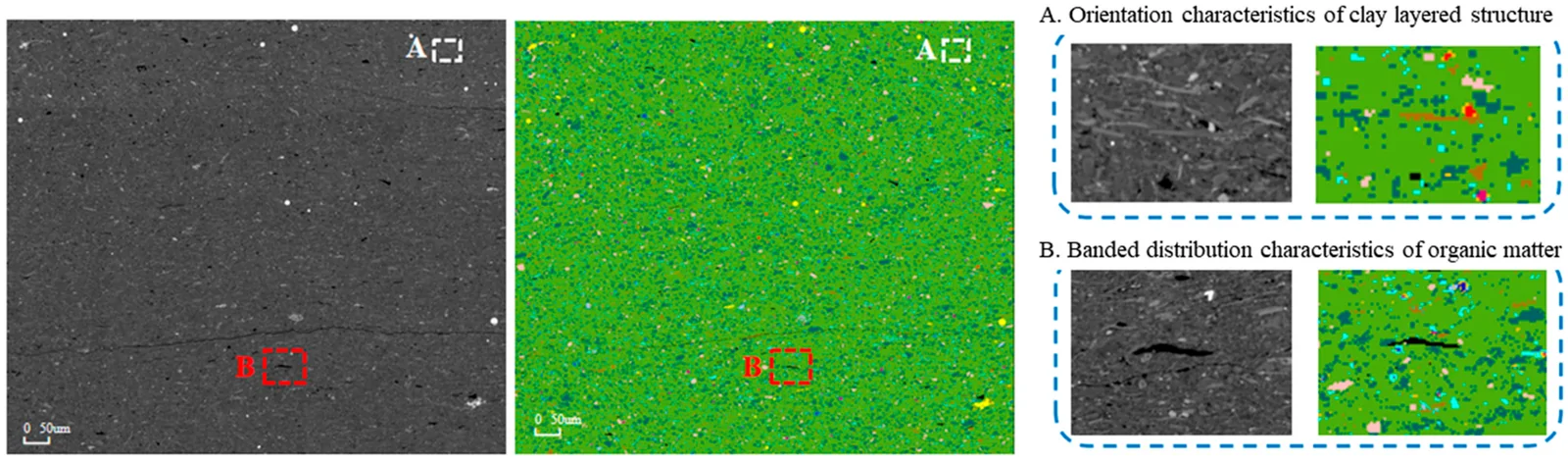
Orientation map of microscale mineral distribution.

**Figure 12:**
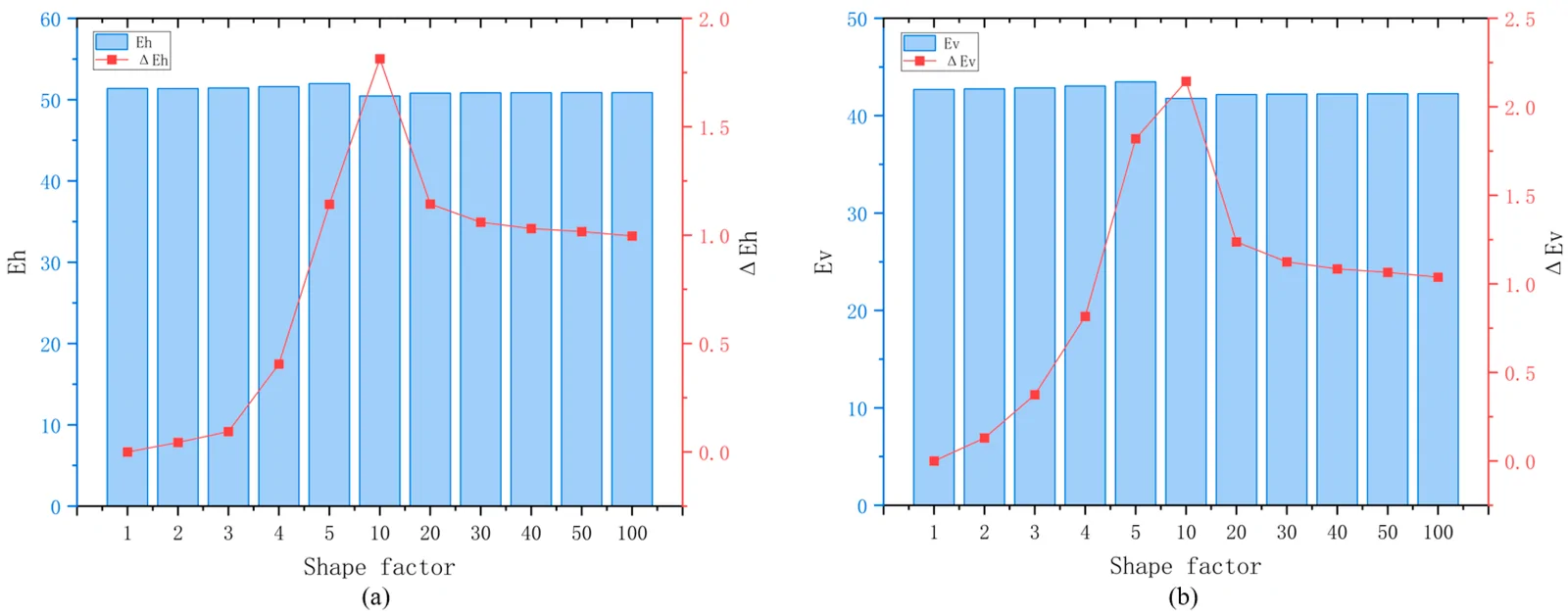
Sensitivity analysis of organic matter (**a**). Parallel to bedding direction; (**b**) bedding-normal direction.

**Table 1:** Elastic parameters of the stiffness matrix for the initial matrix phase.

Elastic Parameters (GPa)	Quartz	Clay
C_11_	72.60	57.30
C_12_	6.22	35.07
C_13_	6.22	30.97
C_33_	72.60	39.32
C_44_	33.18	5.61
C_66_	33.18	11.11

**Table 2:** The Poisson’s ratio for different micro-constituents [42].

Micro-Constituents	Poisson’s Ratio
Pyrite	0.16
Dolomite	0.292
Quartz	0.079
Feldspar	0.285
Calcite	0.309
Clay	0.25
Organic matter	0.25

**Table 3:** Comparison of micromechanical characterization results.

Young’s Modulus (GPa)							Source
Pyrite	Dolomite	Quartz	Feldspar	Calcite	Clay	Organic Matter	
/	87.9–105.	93.9–109.5	69–76.6	61.9–70.9	20.8–39.3	5.8–6.2	[40]
/	97.59–155.50	94.51–143.87	62.17–99.34	67.01–84.05	27.77–35.77	/	[41]
135.48	107.75	71.62	60.95	60.45	21.36	9.13	This work

## Data Availability

The original contributions presented in this study are included in the article. Further inquiries can be directed to the corresponding author.

## Abbreviations

The following abbreviations are used in this manuscript:

ECS	Elemental Capture Spectroscopy
VRH	Voigt–Reuss–Hill
MT	Mori–Tanaka
SEM	Scanning Electron Microscope
EDS	Energy-Dispersive Spectroscopy
XRD	X-ray Diffraction
SPM	Scanning Probe Microscopy
RT	Resistivity
GR	Gamma Ray
AC	Acoustic
DEN	Density
CNL	Neutron
QFM	Quartz and Feldspar
CNN	Convolutional Neural Network
RMSE	Root Mean Square Error
